# Clinical application of parathyroid autotransplantation in endoscopic radical resection of thyroid carcinoma

**DOI:** 10.3389/fonc.2022.942488

**Published:** 2022-08-04

**Authors:** Qi Zhang, Kun-Peng Qu, Ze-Sheng Wang, Jing-Wei Gao, Yu-Peng Zhang, Wei-Jia Cao

**Affiliations:** ^1^ The First Clinical Medical College, Gansu University of Chinese Medicine, Lanzhou, China; ^2^ Department of General Surgery, Gansu Provincial People’s Hospital, Lanzhou, China

**Keywords:** endoscopic radical resection of thyroid carcinoma, parathyroid autotransplantation, transient hypoparathyroidism, permanent hypoparathyroidism, central lymph node dissection

## Abstract

**Purpose:**

This study aimed to examine the effect of selective inferior parathyroid gland autotransplantation on central lymph node dissection(CLND) and incidence of postoperative hypoparathyroidism in patients undergoing endoscopic radical resection of thyroid carcinoma.

**Methods:**

The data of 310 patients undergoing endoscopic radical resection of thyroid carcinoma will be retrospectively analyzed. The patients will be divided into the experimental group and the control group according to whether they combined with parathyroid autotransplantation. Statistics of the incidence rate of postoperative hypoparathyroidism, the concentration of PTH and Calcium in the systemic circulation at different time points in the two groups, the concentration of PTH in the cubital fossa vein in the transplantation region in the experimental group, and the number of central lymph nodes and positive lymph nodes dissection will be carried out.

**Results:**

The incidence rate of temporary and permanent hypoparathyroidism in the experimental group was 33.75% and 0.625%, respectively, and in the control group was 22% and 5%, respectively; its difference was statistically significant (X^2 =^ 10.255, P=0.006). Parathyroid autotransplantation increased incidence of transient hypoparathyroidism (OR, 1.806; Cl, 1.088-2.998; P=0.022), and lower incidence of permanent hypoparathyroidism (OR, 0.112; Cl, 0.014-0.904; P=0.040). The diameters of thyroid cancer nodules was not associated with the occurrence of transient hypoparathyroidism (OR, 0.769; Cl, 0.467-1.265; P=0.301) or permanent hypoparathyroidism (OR, 1.434; Cl, 0.316-6.515; P=0.641). Comparison of systemic circulation PTH, between the two groups showed that the PTH of patients in the experimental group was higher than that in the control group from 1 week to 12 months after the operation, and the difference was statistically significant (P<0.05). In the experimental group, from 1 week to 12 months after surgery, PTH concentrations was significantly higher in the cubital fossa of the transplantation side than in the contralateral side, and the differences were statistically significant (P<0.05). The mean number of central lymph node dissected per patient was significantly higher in the experimental group (7.94 ± 3.03 vs. 6.99 ± 2.86; P <0.05); The mean number of positive nodes per patient was significantly higher in the experimental group (3.16 ± 1.86 vs. 2.53 ± 1.59; P <0.05).

**Conclusions:**

In endoscopic radical resection of thyroid carcinoma, parathyroid autotransplantation is more beneficial to postoperative parathyroid glands function recovery, effectively preventing postoperative permanent hypoparathyroidism and realizing more thorough CLND.

## Introduction

Thyroid cancer is the most common malignant tumor of the endocrine system. Its incidence has been increasing in recent years (1, 2). Bilateral thyroidectomy combined with CLND is a common surgical treatment approach (3). Because of standardization of thyroid disease diagnosis and treatment and an increasing demand for better cosmetic outcomes, more patients and surgeons are selecting endoscopic radical resection, which has become one of the primary surgical methods for thyroid cancer (4, 5). However, this operation is associated with a high risk of parathyroid gland injury. Hypoparathyroidism resulting from damage to the parathyroid gland blood supply or inadvertent parathyroid gland removal can occur, even when the procedure is performed by an experienced specialist (6). Distinguishing the inferior parathyroid glands from enlarged lymph nodes is challenging (7). Retention of the inferior parathyroid glands may limit the extent of thyroidectomy and the thoroughness of CLND, which can lead to postoperative recurrence and metastasis (8). Although in situ gland preservation is one option to preserve parathyroid function, selective inferior gland autotransplantation is another for cases in which the gland is damaged or removed (9). In such cases, we try to preserve all parathyroid glands in situ whenever possible, and when the inferior parathyroid glands are incorrectly incised intraoperatively or when the surgeon judges that their blood supply is impaired, we will selectively transplant one of the inferior parathyroid glands to the non-dominant forearm brachioradialis muscle, leaving the rest of the parathyroid glands in situ, and assess postoperative graft survival by measuring parathyroid hormone (PTH) concentration in venous blood obtained from cubital fossa veins (10, 11). This study aimed to examine the effect of selective inferior parathyroid gland autotransplantation on lymph node dissection and incidence of postoperative hypoparathyroidism in patients undergoing endoscopic radical resection of thyroid carcinoma.

## Method

### Patients

The medical records of all patients who underwent endoscopic radical resection of thyroid carcinoma at Gansu Provincial People’s Hospital from January 2019 to April 2021 were reviewed. All patients underwent preoperative neck ultrasonography, computed tomography, or examination of fine needle aspiration cytology that was consistent with American Thyroid Association (ATA) guidelines for the diagnosis of papillary thyroid carcinoma (PTC) (12). Diagnoses were confirmed by histopathological examination of the surgical specimen. Endoscopic bilateral thyroidectomy was performed for confirmed PTC larger than 1 cm, or older age (>45 years), or with gross extrathyroidal extension, or bilateral foci, or lymph node metastasis, or distant metastasis. We excluded patients with incomplete data, abnormal preoperative parathyroid hormone or calcium concentrations, liver or kidney dysfunction, or a history of neck surgery or radiation therapy. We also excluded those who underwent lateral neck dissection and patients who did not follow up regularly. Patients who underwent endoscopic radical resection of thyroid carcinoma and parathyroid auto transplantation were considered the experimental group. Those who underwent endoscopic radical resection alone were considered the control group.

The study was conducted in accordance with the principles of the Declaration of Helsinki. The study protocol was approved by the Ethics Committee of the Gansu Provincial People’s Hospital(No.2022-195). All patients provided written informed consent.

### Operation methods

**Figure 1:**
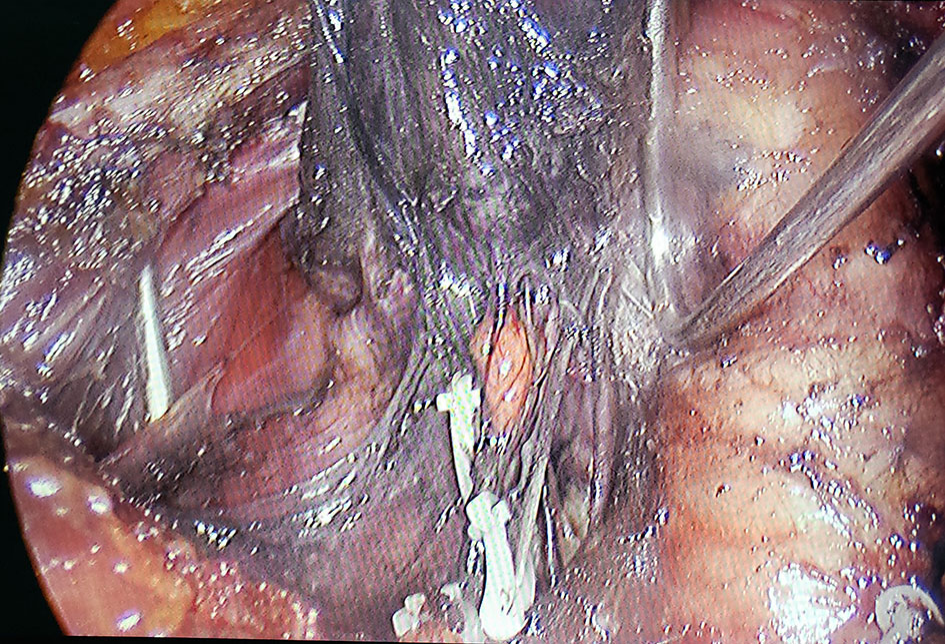
“Negative imaging” of parathyroid glands.

**Figure 2:**
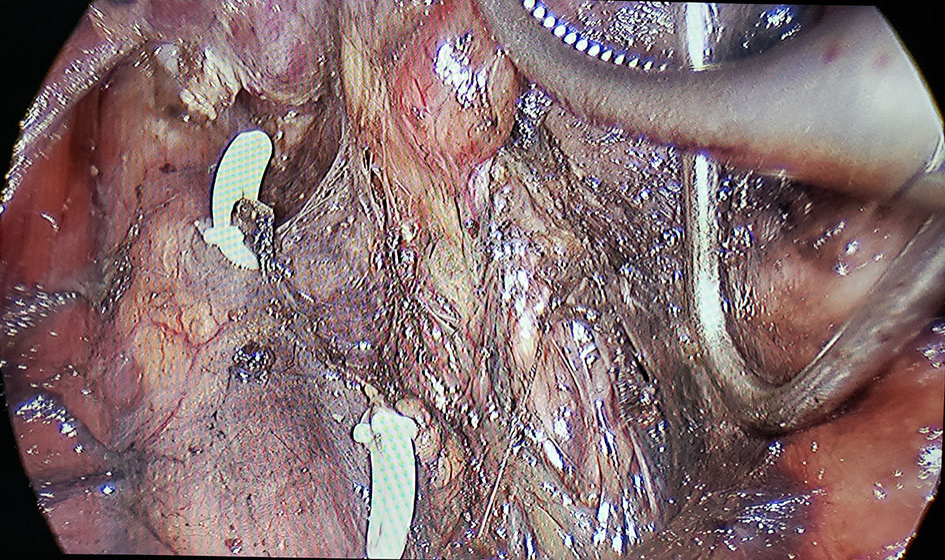
Trachea, nerve, blood vessel, parathyroid, imaging.

**Figure 3:**
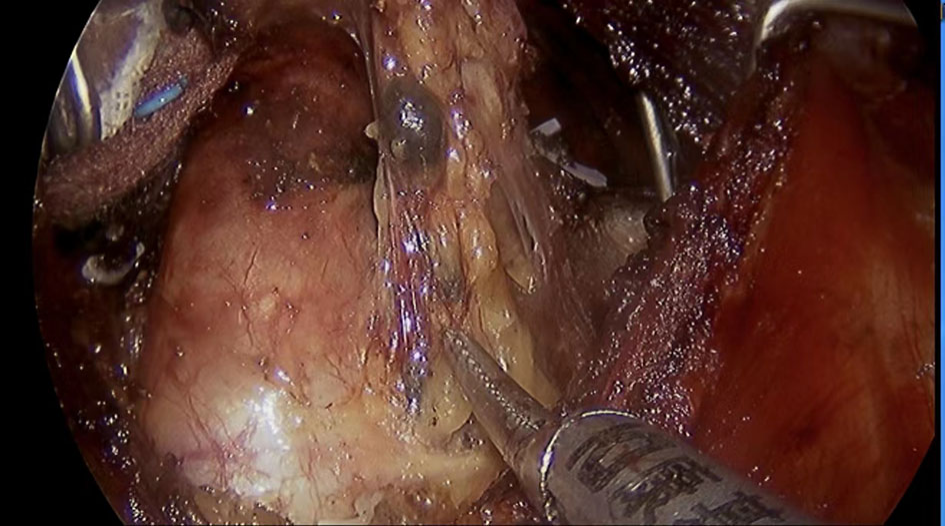
Black-stained central lymph node.

A single surgical team performed all operations. Endoscopic bilateral thyroidectomy and CLND using the complete areola approach were performed. After exposing the thyroid lobes unilaterally, 0.2 mL of a suspension of nano-sized carbon particles was injected percutaneously into the gland (13): the thyroid gland stains black, while the parathyroid gland does not (**Figure 1**). The trachea and recurrent laryngeal nerve were then carefully dissected and the lobes of the thyroid were removed (**Figure 2**). The procedure was repeated contralaterally was performed. For papillary thyroid carcinoma, preventive CLND is routinely performed. (**Figure 3**). Number of central lymph nodes dissected and number of positive lymph nodes were counted. The diameters of thyroid cancer nodules in the two groups after surgery were counted (the diameter of the largest nodule was evaluated in patients with more than one nodule. In the presence of more than one nodule located side by side, the total diameter of the nodules was evaluated.), with 1 cm as the boundary, and the number of patients with thyroid cancer nodules < 1 cm and ≥ 1 cm were counted respectively.

**Figure 4:**
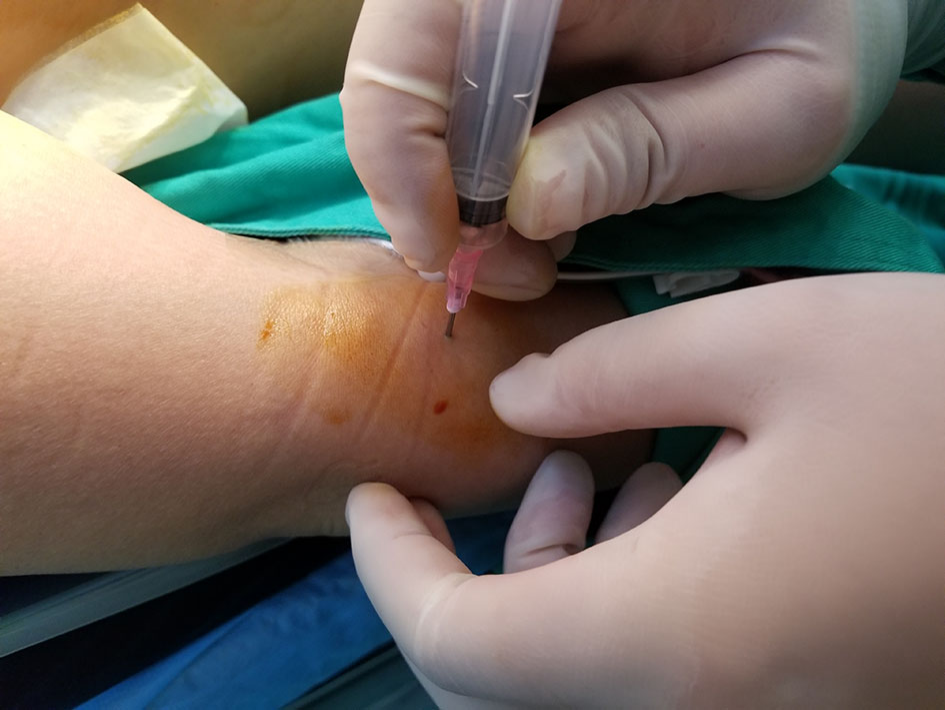
Forearm brachioradialis muscle by homogenate injection.

The superior parathyroid gland is always preserved in situ during surgery, and the inferior parathyroid glands is preserved in situ whenever possible. All parathyroid in situ preservation surgery was used as the control group, and the operation in which the inferior parathyroid gland was incised by mistake or the blood supply was damaged and could not be preserved in situ was set as the experimental group. The surgeon determines the in situ viability of the inferior parathyroid glands and selectively transplants one of the inferior parathyroid gland, this condition is called selective parathyroid autotransplantation. Damaged glands or those removed by mistake were placed in a beaker. After injection of 1 mL 0.9% sodium chloride solution was injected into the tissue, it was cut into pieces and homogenized. The PTH immune gold technique (14) was used to confirm parathyroid tissue. The gland suspension was then implanted into the brachioradialis muscle of the non-dominant forearm via injection (**Figure 4**).

### Postoperative protocol

After the operation, calcium and calcitriol supplements were administered (15) and systemic PTH and calcium concentrations were measured. If both were in the normal range (PTH, 16–88 pg/mL; calcium, 2.11–2.52 mmol/L), calcium supplementation was ceased. Hypoparathyroidism was defined as PTH <16 pg/mL or calcium <2.11 mmol/L. Permanent hypoparathyroidism was defined as hypoparathyroidism that persisted >6 months after surgery. According to the 2015 American Thyroid Association (ATA) guidelines (12), Routinely reviewing serum thyroglobulin (Tg), serum thyroglobulin antibodies (Tg-ab) (serum Tg and Tg-ab should be assessed longitudinally in the same laboratory and using the same assay for a given patient)and neck ultrasound after the operation to assess the risk of postoperative disease recurrence. Systemic PTH and calcium concentration were measured before and 1 day, 1 week, 1 month, 3 months, 6 months, and 12 months after surgery. In the experimental group, PTH concentration was measured in venous blood sampled from a cubital fossa vein adjacent to the transplantation site before and 1 day, 1 week, 1 month, 3 months, 6 months, and 12 months after surgery. These measurements were compared with those taken from the cubital fossa contralateral to the parathyroid transplantation site.

### Statistical analysis

Statistical analyses were performed using SPSS software version 22.0 (IBM Corp., Armonk, NY, USA). Categorical data are expressed as numbers with percentage and were compared using the chi-square or Fisher’s exact test. Continuous data with a normal or near-normal distribution are expressed as means with standard deviation and were compared using the independent sample t test or corrected t test, depending on group variance. Risk factors for transient and permanent hypoparathyroidism were identified using logistic regression with results presented as odds ratios (ORs) with 95% confidence interval (CI). P <0.05 was considered significant.

## Results

### General data

In total, 310 patients were included for analysis, 160 in the experimental group and 150 in the control group. The experimental group included 21 men and 139 women with mean age 43.68 ± 10.48 years (range, 22–77). In the control group, 26 were men and 124 were women; mean age was 43.01 ± 11.33 years (range, 20–72). As shown in **Table 1**, patient characteristics did not significantly differ between the groups.

**Table 1:** General data, surgical biochemical data and postoperative pathology of patients.

Grouping	Experimental group	Control Group	t/X^2^	P
Age	43.68 ± 10.48	43.01 ± 11.33	T = 0.534	0.594
Gender			X^2 ^= 1.066	0.302
Male	21 (13.13%)	26 (17.33%)		
Female	139 (86.87%)	124 (82.67%)		
Hypoparathyroidism after operation			X^2 ^= 10.255	0.006
Transient hypoparathyroidism	54 (33.75%)	33 (22%)		
Permanent hypoparathyroidism	1 (0.625%)	8 (5%)		
PTH (pg/mL, $\bar{X}\pm \text{S}$ )				
Per	58.67 ± 12.33	57.35 ± 11.28	T = 0.978	0.329
Post 1 day	19.05 ± 6.05	19.48 ± 6.12	T = 0.622	0.535
Post 1 week	24.59 ± 6.68	20.06 ± 6.12	T = 6.211	0.000
Post 1 month	37.71 ± 13.32	28.87 ± 9.36	T = 6.787	0.000
Post 3 months	47.76 ± 13.71	34.03 ± 10.01	T = 10.110	0.000
Post 6 months	48.80 ± 13.35	37.55 ± 9.81	T = 8.490	0.000
Post 12 months	49.73 ± 14.76	39.65 ± 9.78	T = 7.127	0.000
Ca^2+^ (mmol/L, $\bar{X}\pm \text{S}$ )				
Per	2.33 ± 0.09	2.32 ± 0.08	T = 0.343	0.732
Post 1 day	2.16 ± 0.11	2.14 ± 0.07	T = 1.871	0.062
Post 1 week	2.17 ± 0.05	2.16 ± 0.05	T = 1.426	0.155
Post 1 month	2.23 ± 0.06	2.20 ± 0.07	T = 4.953	0.000
Post 3 months	2.26 ± 0.06	2.23 ± 0.06	T = 4.862	0.000
Post 6 months	2.32 ± 0.06	2.26 ± 0.08	T = 6.421	0.000
Post 12 months	2.32 ± 0.06	2.29 ± 0.08	T = 3.804	0.000
The diameters of thyroid cancer nodules	0.94 ± 0.41	0.91 ± 0.43	T = 0.529	0.597
Lymph nodes dissection				
Number of central lymph nodes dissection	7.94 ± 3.03	6.99 ± 2.86	T = 2.833	0.005
Number of positive lymph nodes	3.16 ± 1.86	2.53 ± 1.59	T = 3.160	0.002

Note: Per, per-operative; Post, post-operative.

### Hypoparathyroidism data

A total of 55 patients in the experimental group developed hypoparathyroidism: 54 cases (33.75%) were transient and one (0.625%) was permanent. The single permanent hypoparathyroidism patient recovered normalized PTH secretion 12 months after surgery. In the control group, 39 patients developed hypoparathyroidism: 33 cases (22%) were transient and 8 cases (5%) were permanent. Four of the Eight permanent cases recovered normal PTH secretion 12 months after surgery. The incidence of transient hypoparathyroidism was higher in the experimental group than in the control group; however, the incidence of permanent hypoparathyroidism was higher in the control group. The difference between groups was significant (X2 = 10.255; P = 0.006; **Table 1**). In univariate analysis, parathyroid autotransplantation increased incidence of transient hypoparathyroidism (OR,1.806;Cl,1.088-2.998;P=0.022),and lower incidence of permanent hypoparathyroidism (OR,0.112;Cl,0.014-0.904;P=0.040) (**Table 2**).

**Table 2:** Parathyroid autotransplantation and risk of hypoparathyroidism.

Grouping	Experimental group	Control Group	OR (95%Cl)	P
Transient hypoparathyroidism	54 (33.75%)	33 (22%)	1.806 (1.088-2.998)	0.022
Permanent hypoparathyroidism	1 (0.625%)	8 (5%)	0.112 (0.014-0.904)	0.040

### Systemic PTH and calcium concentrations before and after surgery

Systemic PTH concentration did not significantly differ between the groups before surgery. After surgery, PTH concentration was significantly higher in the experimental group, except on day 1 (**Table 1**). In the experimental group, PTH concentration significantly differed between before surgery and 1 day after, 1 day and 1 week after, 1 week and 1 month after, and 1 month and 3 months after surgery(P<0.05); the concentration did not significantly differ between 3 months and 6 months after surgery(P>0.05). In the control group, PTH concentration did not significantly differ between 6 months and 12 months after surgery(P<0.05) (**Table 3**). Six months after surgery, PTH secretion in the experimental and control groups had recovered to 83.16% and 65.48% of preoperative baseline, respectively.

**Table 3:** Comparing the PTH between the two time points of the same group(pg/mL, X±S)

time point	Experimental group			Control Group		
	PTH	T	P	PTH	T	P
Per VS Post 1 day	(58.67 ± 12.33) VS (19.05 ± 6.05)	36.479	0.000	(57.35 ± 11.28) VS (19.48 ± 6.12)	36.131	0.000
Post 1 day VS Post 1 week	(19.05 ± 6.05) VS (24.59 ± 6.68)	7.772	0.000	(19.48 ± 6.12) VS (20.06 ± 6.12)	0.821	0.412
Post 1 week VS Post 1 month	(24.59 ± 6.68) VS (37.71 ± 13.32)	11.133	0.000	(20.06 ± 6.12) VS (28.87 ± 9.36)	9.654	0.000
Post 1 month VS Post 3 months	(37.71 ± 13.32) VS (47.76 ± 13.71)	6.650	0.000	(28.87 ± 9.36) VS (34.03 ± 10.01)	4.611	0.000
Post 3 months VS Post 6 months	(47.76 ± 13.71) VS (48.80 ± 13.35)	0.690	0.491	(34.03 ± 10.01) VS (37.55 ± 9.81)	3.076	0.002
Post 6 months VS Post 12 months	(48.80 ± 13.35) VS (49.73 ± 14.76)	0.588	0.557	(37.55 ± 9.81) VS (39.65 ± 9.78)	1.851	0.065

Note: Per, per-operative; Post, post-operative.

Systemic calcium concentration did not significantly differ between the groups before surgery. After surgery, calcium concentration was significantly higher in the experimental group, except on day 1 and week 1 (**Table 1**).

### Postoperative specimens

In the experimental and control groups, the number of central lymph nodes dissected was 1271 and 1049, respectively. The mean number of central lymph node dissected per patient was significantly higher in the experimental group (7.94 ± 3.03 vs. 6.99 ± 2.86; P <0.05). The number of positive lymph nodes in the experimental and control groups was 505 and 380, respectively. The mean number of positive nodes per patient was significantly higher in the experimental group (3.16 ± 1.86 vs. 2.53 ± 1.59; P <0.05; **Table 1**). The average diameter of thyroid cancer nodules in the experimental group was (0.94 ± 0.41) cm, and that in the control group was (0.91 ± 0.43) cm. There wasdid not significantly differ in the average diameter of thyroid cancer nodules between the two groups (P>0.05) (**Table 1**). There were 150 patients with the diameters of thyroid cancer nodules ≥1cm in the two groups, 38 patients had transient hypoparathyroidism after surgery, and 4 patients had permanent hypoparathyroidism. There were 160 cases of the diameters of thyroid cancer nodules <1cm, 49 cases of transient hypoparathyroidism after operation, 3 cases of permanent hypoparathyroidism. In univariate analysis, The diameters of thyroid cancer nodules was not associated with the occurrence of transient hypoparathyroidism (OR, 0.769; Cl, 0.467-1.265; P=0.301) or permanent hypoparathyroidism (OR, 1.434; Cl, 0.316-6.515; P=0.641) (**Table 4**).

**Table 4:** The diameters of thyroid cancer nodules and risk of hypoparathyroidism.

Grouping	Diameter ≥ 1cm	Diameter <1 cm	OR (95%Cl)	P
Transient hypoparathyroidism	38 (25.3%)	49 (30.6%)	0.769 (0.467-1.265)	0.301
Permanent hypoparathyroidism	4 (2.6%)	3 (1.88%)	1.434 (0.316-6.515)	0.641

### Postoperative cubital fossa venous PTH concentration in the experimental group

In the experimental group, cubital fossa venous blood PTH concentration did not significantly differ between the transplantation side and the contralateral side 1 day after surgery (P>0.05). From 1 week to 12 months after surgery, serum PTH in the transplantation region was significantly higher than that in the non-transplantation region, and the differences were statistically significant (P<0.05). PTH concentration increased in both the transplantation and contralateral sides as time progressed to a peak at 6 months; then, the concentration began to decline (**Table 5**).

**Table 5:** Comparison between cubital fossa vein PTH in transplantation region and non-transplantation region in the experimental group (pg/mL, X±S).

Grouping	N	Post 1 day	Post 1 week	Post 1 month	Post 3 months	Post 6 months	Post 12 months
Transplantation region	160	20.46 ± 7.45	34.39 ± 10.73	83.77 ± 21.86	99.15 ± 22.92	105.48 ± 22.31	83.53 ± 15.80
Non-transplantation region	160	19.05 ± 6.05	24.39 ± 6.68	37.71 ± 13.32	47.76 ± 13.71	48.79 ± 13.38	49.67 ± 14.89
T		1.862	9.806	22.757	24.345	27.569	19.726
P		0.064	0.000	0.000	0.000	0.000	0.000

Post, post-operative.

## Discussion

Hypoparathyroidism is a common complication of endoscopic radical resection of thyroid carcinoma (16) and may be transient or permanent. In the former, transient hypocalcemia is treated using calcium supplementation and patient quality of life is not significantly affected. The latter causes permanent hypocalcemia, which may lead to abnormal calcification in the brain, kidney, eyes, blood vessels, and other systems (17, 18), which will seriously increase patients’ disease and economic burden. The incidence of hypoparathyroidism after thyroid surgery varies considerably (19). Reported incidence rates of transient hypoparathyroidism range from 5.49% to 67.69%; those for permanent hypoparathyroidism range from 0% to 20% (16, 20, 21). This problem is caused by a number of factors (22, 23), one of which is that the definition of the timing of hypoparathyroidism is still being explored. The guidelines of the European Society of Endocrinology (24) and the ATA (25) define permanent hypoparathyroidism as a low PTH concentration 6 months after surgery. However, Kim et al. (26) showed that some patients diagnosed with permanent hypoparathyroidism eventually recover normal PTH secretion. Qiu et al. (27) found that 45.2% of patients were misdiagnosed with permanent hypoparathyroidism when 6 months was used as the cutoff point to define permanence. In our study, the one patient (100%) in the experimental group who developed permanent hypoparathyroidism eventually recovered normal PTH secretion 12 months after surgery. In the control group, four of the eight patients who developed it (50%) recovered normal secretory function at 12 months. The reason for the difference in functional recovery between groups is probably related to the lower surface area of the transplanted gland compared with the in situ gland: the angiogenic response to establish gland perfusion is more robust and rapid for a gland with lower surface area (28). Parathyroid glands’ surface area in the transplantation region was lesser, which was more likely to occur angiogenic reactions with surrounding tissues, realizing the reconstruction of the blood supply system. However, in-situ reserved parathyroid glands in the control group did not have a normal secretory function, their body surface area was larger, and the angiogenesis was slower, so the parathyroid glands’ function recovered in the control group was more complicated than that in the experimental group. Previous studies have shown that parathyroid functional recovery is a dynamic process that may occur over a long period after thyroid surgery (29).

There is a general consensus that parathyroid autotransplantation during thyroidectomy increases the risk of transient hypoparathyroidism, but the effect on permanent hypoparathyroidism remains unclear. This is the main controversy of whether to perform parathyroid autotransplantation in thyroidectomy. Ahmed et al. (30) proposed that autologous parathyroid transplantation is the preferred method to ensure good parathyroid function and recommended routine transplantation. Qiu et al. (31) concluded that parathyroid autotransplantation is an independent risk factor for transient hypoparathyroidism but a preventive factor for permanent hypoparathyroidism and recommended selective parathyroid autotransplantation. Su et al. (32) found that parathyroid autotransplantation during thyroidectomy was not associated with the development of permanent postoperative hypoparathyroidism, so it is recommended that parathyroid glands be preserved in situ whenever possible. Some experts (29) even suggest that parathyroid autotransplantation may increase the risk of permanent postoperative hypoparathyroidism. We do not recommend routine parathyroid autotransplantation, but believe that selective parathyroid autotransplantation is the most effective way to salvage injured parathyroid function in cases of intraoperative miscut or injury to avoid permanent postoperative hypoparathyroidism. In our study, the incidence of transient hypoparathyroidism was higher in the experimental group than in the control group (33.75% vs. 22%). However, the incidence of permanent hypoparathyroidism was lower in the experimental group (0.625% vs. 4%); The differences were significant (X2 = 8.327; P = 0.013). In univariate analysis, parathyroid autotransplantation increased incidence of transient hypoparathyroidism (OR,1.806;Cl,1.088-2.998;P=0.022),and lower incidence of permanent hypoparathyroidism (OR,0.151;Cl,0.018-1.269;P=0.046). Selective parathyroid autotransplantation during endoscopic radical resection of thyroid carcinoma is effective in preventing permanent hypoparathyroidism. This is consistent with the findings of Qiu’s (31) study. At the same time, we found that the diameters of thyroid cancer nodules was not associated with the occurrence of transient hypoparathyroidism or permanent hypoparathyroidism which was the same conclusion as Yazıcıoğlu et al (22).

Currently, parathyroid function after thyroid surgery is evaluated using clinical symptoms and serum PTH and calcium concentrations (33). In previous studies, the sternocleidomastoid muscle was selected as the graft site to avoid making a second surgical incision; however, this method precludes differentiating parathyroid function in the transplantation site and the preserved parathyroid glands (34, 35). Therefore, we selected the brachioradialis in the non-dominant forearm as the graft site. Graft survival was determined when the PTH concentration ratio of the cubital fossa vein in both arms reached 1.5 times (36). One previous study (37) that examined transplantation sites reported that graft function assessment is easiest when the brachioradialis is used and that use of this site is safe and effective. PTH concentration decreased on the first day after surgery in both groups. Since the function of the parathyroid glands retained in-situ could not be wholly predicted, in the experimental group, the transplanted gland required time to establish perfusion (28), the graft had no secretory ability on the first postoperative day, and only the retained in-situ and well-functioning part of the parathyroid glands in both groups secreted PTH. Furthermore, a dilution effect likely occurred in both groups owing to postoperative fluid supplementation. These factors resulted in a significant decrease in serum PTH concentration in the experimental and control groups 1 day after surgery. Systemic PTH concentrations was significantly higher in the experimental group from 1 week to 12 months after surgery. During this time period, PTH concentrations was significantly higher in the cubital fossa of the transplantation side than in the contralateral side, suggesting that the transplanted parathyroid glands survived and began to secrete PTH within a week. This is similar to the findings of Zhang et al. (11), who found that 96.5% demonstrated the first evidence of graft function within 2 weeks, and 3.5% showed graft function 2-8 weeks postoperatively. The mean interval to parathyroid autograft functioning was 1.3 ± 0.9 weeks. However, El-Sharaky (38) observed using electron microscopy for 4 weeks and concluded that the transplanted parathyroid glands became secretory by the second postoperative week and approached a normal state by 4 weeks postoperatively. We found that as the time from transplantation increased, PTH secretory function of the transplanted parathyroid glands increased. Optimal secretion function was reached 6 months after surgery, and then began to gradually decrease to normal by 12 months. We hypothesize that, on the one hand, normal secretory function was restored in the parathyroid glands preserved in situ and the transplanted parathyroid function was inhibited by negative feedback. On the other hand, the body has changed from the compensatory state of the parathyroid glands after the operation to a normal physiological process, whether in-site retained or transplanted parathyroid glands could secrete PTH according to the needs of the body.

By the comparison between 2-time points of the same group, PTH concentration did not significantly differ in the experimental group between 3 months and 6 months after surgery nor in the control group between 6 months and 12 months after surgery. These findings show that functional parathyroid recovery in the experimental group was basically stable 6 months after surgery. In contrast, stable recovery in the control group was not reached until 12 months. Autotransplantation may be more conducive to enabling earlier functional recovery. Qiu et al. (27) also reported the same conclusion. Finally, PTH secretory function recovered to 83.16% of baseline in the experimental group, but only 65.48% of baseline in the control group, further suggesting that autotransplantation is more conducive for postoperative functional recovery. However, parathyroid function remained below baseline in both groups. We speculate that, on the one hand, damaged or disconnected parathyroid glands may have been retained in situ during the operation but their secretory function was impaired. On the other hand, the graft may have lost its secretory function because of insufficient blood supply or fibrosis (26).

Calcium concentration was significantly higher in the experimental group than in the control group 1, 3, 6, and 12 months after surgery. By analyzing the changes in serum PTH and Calcium concentrations in the two groups after the operation, we found that PTH and Calcium increased after the operation did not maintain absolute consistency but existed separation phenomenon. We speculate that PTH mainly increases calcium concentration *via* two mechanisms (39): (1) PTH enhances osteoclast activity to promote release of calcium phosphate into the blood; and (2) PTH enhances calcium reabsorption in renal tubules and stimulates 1,25(OH)2 D3 production in the kidney to affect intestinal calcium absorption. There was a time lag between recovery of serum PTH and calcium concentrations, suggesting that calcium homeostasis mechanisms required time to establish. Furthermore, routine calcium and calcitriol supplementation after surgery caused postoperative calcium testing to be an inaccurate reflection of PTH secretion by the patient. Normalization of postoperative serum calcium concentration with supplementation may have a benefit, as we hypothesize that it puts injured and transplanted parathyroid tissue into a quiescent state, enabling more rapid and robust postoperative recovery of perfusion and function (40). Goltzman et al. (41) found that increased serum calcium and 1,25(OH)2 D3 concentrations can inhibit the transcription and stability of the PTH gene and that sustained hypercalcemia can inhibit parathyroid cell proliferation and reduce volume of functional parathyroid tissue. Therefore, we suggest that postoperative calcium supplementation should be tailored based on biochemical indicators.

The role of preventive CLND in PTC resection is controversial. Although the ATA (12) and National Comprehensive Cancer Network guidelines (42) do not recommend routine dissection, the Japanese Association of Endocrine Surgeons does (43). A recent retrospective study (44) evaluated preventive CLND in patients with PTC and found that preventive CLND can prevent recurrence and improve disease-free survival in patients at intermediate and high risk of recurrence. Therefore, we routinely perform CLND. In our study, the mean numbers of lymph nodes dissected per patient and positive lymph nodes per patient were significantly higher in the experimental group. Analyze the reasons, the inferior parathyroid glands arise from the third pharyngeal pouch based on anatomy (45). Therefore, the location of the inferior parathyroid glands is highly variable, and it is more common in the area between the lower level of the thyroid gland and the thymus. This makes it difficult to differentiate the inferior parathyroid glands from enlarged lymph nodes. In the control group, when the CLND was performed, the operator was relatively conservative in order to protect the inferior parathyroid glands, the scope of the operation was limited, and the number of lymph nodes dissected was limited. In the experimental group, when the CLND was performed, since the inferior parathyroid gland had been transplanted, the operator had no fear of the inferior parathyroid glands being miscut or damaged to the blood supply in this area, so a more thorough and aggressive lymph node dissection could be performed. Therefore, the number of lymph nodes dissected in the central region was more than that in the control group. We conclude that inferior parathyroid gland autotransplantation enables surgeons to achieve more comprehensive CLND, which may reduce the probability of reoperation. This is consistent with the results reported by Wei et al. (8). All patients in our study were followed for 12 months after surgery and no recurrence or metastasis was found. The characteristically slow progression of thyroid cancer and our relatively short follow-up time and small sample size may explain the low recurrence and metastasis rates.

This study has several limitations. First, as a retrospective study, selection bias may have been introduced. Second, the operator’s judgment of parathyroid function was subjective to an extent; development of technology that can objectively evaluate parathyroid blood supply is warranted. Near-infrared fluorescent/indocyanine green (NIR/ICG) fluorescence imaging technology is a new research field. After ICG is injected into the human body, the tissue structure can be visualized by NIR. Studies have shown that parathyroid glands with good blood supply can take up ICG imaging, and the fluorescence intensity is positively correlated with the dose of ICG (46). Therefore, the imaging intensity can be used to evaluate the blood supply of the parathyroid glands, so as to evaluate the function of the parathyroid glands and decide whether to perform parathyroid autotransplantation. Its application prospect is worth looking forward to.

## Conclusions

Selective inferior parathyroid autotransplantation is effective to prevent permanent hypoparathyroidism when performing endoscopic radical resection of thyroid cancer. However, it is associated with a risk of transient hypoparathyroidism. Strategic transplantation of damaged inferior parathyroid glands or those with poor blood supply is more conducive to early functional recovery of parathyroid secretory function than in situ preservation. Moreover, autotransplantation allows a more thorough CLND, which may reduce recurrence.

## Data availability statement

The original contributions presented in the study are included in the article/supplementary material. Further inquiries can be directed to the corresponding author.

## Ethics statement

The studies involving human participants were reviewed and approved by Ethics Committee of the Gansu Provincial People’s Hospital. The patients/participants provided their written informed consent to participate in this study. Written informed consent was obtained from the individual(s) for the publication of any potentially identifiable images or data included in this article.

## Author contributions

QZ and K-PQ conceived and designed the research. Z-SW and J-WG collected the data and conducted the research. Y-PZ and W-JC analyzed and interpreted the data. QZ wrote the initial paper. K-PQ revised the paper. QZ and K-PQ had primary responsibility for the final content. All authors contributed to the article and approved the submitted version.

## Funding

This work was supported by following grants: Natural Science Foundation of Gansu Province of China(145RJZA116); Special Fund for Clinical Research of Wu Jieping Medical Foundation (320.6750.16216)

## Conflict of interest

The authors declare that the research was conducted in the absence of any commercial or financial relationships that could be construed as a potential conflict of interest.

## Publisher’s note

All claims expressed in this article are solely those of the authors and do not necessarily represent those of their affiliated organizations, or those of the publisher, the editors and the reviewers. Any product that may be evaluated in this article, or claim that may be made by its manufacturer, is not guaranteed or endorsed by the publisher.
